# Changes in the prevalence of diabetes and control of risk factors for diabetes among Chinese adults from 2007 to 2017: An analysis of repeated national cross‐sectional surveys

**DOI:** 10.1111/1753-0407.13492

**Published:** 2023-11-05

**Authors:** Chenye Jin, Yaxin Lai, Yongze Li, Di Teng, Wenying Yang, Weiping Teng, Zhongyan Shan, For the China National Diabetes and Metabolic Disorders Study Group and the Thyroid Disorders, Iodine Status and Diabetes Epidemiological Survey Group

**Affiliations:** ^1^ Department of Rheumatology and Immunology First Hospital of China Medical University Shenyang China; ^2^ Department of Endocrinology and Metabolism and the Institute of Endocrinology, NHC Key Laboratory of Diagnosis and Treatment of Thyroid Diseases First Hospital of China Medical University Shenyang China; ^3^ Department of Endocrinology China‐Japan Friendship Hospital Beijing China

**Keywords:** diabetes, epidemiology, prediabetes, prevalence

## Abstract

**Introduction:**

To examine changes in the prevalence of diabetes and the control of risk factors for diabetes over 10 years among adults in China.

**Methods:**

Two population‐based cross‐sectional surveys were used to obtain a nationally representative sample of adults aged 20 years and older in mainland China in 2007 (*n* = 46 239) and 2017 (*n* = 73 340). Changes in the prevalence of diabetes, impaired fasting glucose, impaired glucose tolerance, and prediabetes, as diagnosed by the World Health Organization criteria, were assessed over time.

**Results:**

The weighted prevalence of diagnosed diabetes (3.8% vs 6.3%, *p* = .0001) and total diabetes (9.7% vs 11.7%, *p* = .005) increased among the overall population between 2007 and 2017. The weighted prevalence of undiagnosed diabetes (5.9% vs 5.4%, *p* = .7), impaired fasting glucose (2.7% vs 2.6%, *p* = .68), impaired glucose tolerance (12.7% vs 12.5%, *p* = .95), prediabetes (15.4% vs 15.1%, *p* = .79), the treatment of diabetes (34.1% vs 32.5%, *p* = .44), and the control of diabetes (31.1% vs 32.8%, *p* = .73) did not significantly change over this period. The awareness of diabetes (39.4% vs 53.6%, *p* = .0004) increased over 10 years among the overall population. The proportion of achieved high‐density lipoprotein cholesterol targets increased (*p* = .005), but the proportion of achieved body mass index (*p* = .01) and waist circumference (*p* = .0002) targets decreased significantly.

**Conclusions:**

Between 2007 and 2017, the prevalence of total diabetes (diagnosed by the World Health Organization criteria), especially diagnosed diabetes, increased among adults in China. Although awareness of diabetes improved, effective interventions and clinical strategies are urgently required.

## Graphical abstract

**Highlights**

- The prevalence of total diabetes and diagnosed diabetes significantly increased among adults in China between 2007 and 2017
- Waist circumference and the lipid accumulation product index were significantly increased among the overall and diabetic populations
- Only one fifth of patients diagnosed with diabetes had achieved all eight risk factor control goals in 2017

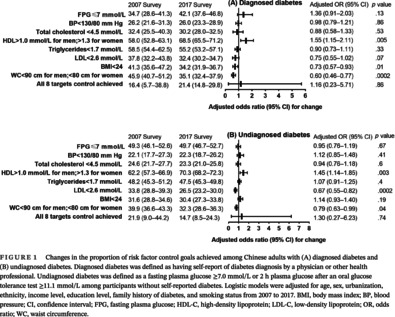

## 1 INTRODUCTION

Diabetes has become a prominent public health issue. An estimated 537 million adults worldwide are currently living with diabetes, and this number is predicted to rise to 783 million by 2045, as reported by the International Diabetes Federation in 2021.^1^ China, as the most populated country, has the largest number of adults with diabetes. This figure has been projected to increase from 140.9 million in 2021 to 174.4 million in 2045.^1^ China has ranked first in the world in the number of residents with diabetes for more than 40 years, and the global proportion of the number of people with diabetes increased from 18.9% in 1980 to 24.4% in 2014.^2^ Diabetes complications and associated deaths have contributed to annual diabetes‐related health care expenditures in China, which ranks second in the world on these expenditures at $165.3 billion.^1^ The rapid development of the Chinese economy in the past 3 decades has led to changes in lifestyle among adults that have increased the national prevalence of diabetes based on the World Health Organization diagnostic criteria from 0.67% in 1980 to 11.9% in 2018.^3^, ^4^

Diabetes prevalence has important implications for the allocation of diabetes management resources. An understanding of the changes in diabetes‐related metrics over time can guide future prevention strategies. Previous studies have reported an increasing prevalence of diabetes among Chinese adults.^5^, ^6^, ^7^, ^8^ However, most of these surveys were limited to specific subpopulations or employed samples that were not nationally representative. To our knowledge, only one nationally representative study with a consistent study design has documented an increase in the prevalence of diabetes in China from 2013 to 2018.^4^ Dynamic changes in population structure, lifestyles, education levels, and economic development should be considered when assessing changes in prevalence over time. Furthermore, appropriate management of well‐established risk factors such as blood pressure, blood glucose levels, and blood lipid levels is crucial to prevent cardiovascular disease in diabetic patients.^9^, ^10^ However, it is unclear whether the control of risk factors in diabetic patients has changed over the past decade. Furthermore, assessments with longer intervals are essential to determine the effects of public health policies and delivery of medical care.

Thus, this analysis had two aims: the primary objectives were to assess changes in the prevalence of diabetes and prediabetes, as well as awareness, treatment, and control rates of diabetes in mainland China over 10 years (from 2007 to 2017). This was accomplished by using nationally representative data from two population‐based cross‐sectional surveys after adjusting for confounding sociodemographic factors. The secondary objectives were to determine the changes in the control of risk factors for diabetes among Chinese adults.

## 2 MATERIALS AND METHODS

### 2.1 Study design

The first nationwide cross‐sectional study included in this analysis was conducted in mainland China between 2007 and 2008 to assess the prevalence of diabetes and metabolic disorders among Chinese adults. Details of the study design are described elsewhere.^11^ Briefly, we employed a multistage stratified random sampling approach to select a nationally representative sample of adults aged 20 and older from mainland China (Supplementary Figure S1). The second cross‐sectional survey was conducted between 2015 and 2017. The details of the study design are provided elsewhere, but a detailed flow chart of the study design is presented in Supplementary Figure S1.^12^, ^13^ In brief, an identical multistage stratified random sampling method was applied in urban and rural areas to obtain a nationally representative sample (Supplementary Figure S1). The inclusion criteria for this study were participants aged 20 years or older who were not pregnant. In total, 46 239 participants in 2007 and 73 340 participants in 2017 were eligible for inclusion, after the exclusion of participants with missing information on sex, age, or blood glucose levels (Supplementary Figure S1). All participants provided written informed consent and the study was conducted according to the Declaration of Helsinki. The research protocols were approved by the Medical Ethics Committees of China Medical University and China–Japan Friendship Hospital. Written informed consent was obtained from all participants.

### 2.2 Data collection

Trained and qualified interviewers used detailed questionnaires to collect information for each participant, including demographic characteristics, behavioral data, and personal medical history. Current smokers were identified as individuals who currently smoked and who had smoked at least 100 cigarettes in their lifetime. Body mass index (BMI) was defined as weight (kg) divided by height (meters) squared. Blood pressure, weight, height, and waist circumference (WC) were measured using standard methods.^13^ Levels of blood glucose, serum total cholesterol, high‐density lipoprotein cholesterol (HDL‐C), low‐density lipoprotein cholesterol (LDL‐C), and triglycerides were measured after an overnight fast of at least 10 h for every participant and at 2 h after a 75 g oral glucose tolerance test among participants without a self‐reported diabetes history. Blood glucose level, serum total cholesterol, and triglycerides were measured with the use of an enzymatic method. All these laboratory tests were successfully completed following a standard protocol.^11^, ^12^ The lipid accumulation product (LAP) index was calculated from the fasting triglyceride levels (mmol/L) and WC (cm) as follows: LAP = (WC‐65) × triglycerides for men; LAP = (WC‐58) × triglycerides for women.^14^

### 2.3 Health outcomes

The diagnostic criteria for diagnosed diabetes, undiagnosed diabetes, total diabetes, impaired fasting glucose (IFG), impaired glucose tolerance (IGT), prediabetes, and control of diabetes are listed in Supplementary Table S1.^15^ According to the Chinese Diabetes Society guidelines for diabetes control and treatment, control of clinical risk factors was specified as follows: fasting plasma glucose ≤7.0 mmol/L, blood pressure < 130/80 mmHg; total cholesterol <4.5 mmol/L; HDL‐C > 1.0 mmol/L for men or HDL‐C > 1.3 mmol/L for women; triglycerides <1.7 mmol/L; LDL‐C < 2.6 mmol/L; and BMI <24 kg/m^2^ among adults with diagnosed diabetes.^16^ The control rate of WC (<90 cm for men and < 80 cm for women) was also estimated.^17^

### 2.4 Statistical analysis

The same statistical method was used to interpret the complex sampling designs of the two surveys to obtain prevalence estimates and 95% confidence intervals (CIs) according to the Taylor linearization method.^18^ The weighted estimates reflect the urban–rural distribution of adults living in mainland China by age, sex, and geographic region. The weighting coefficients were obtained from the Chinese population census data. Categorical variables were expressed as percentages and 95% CIs and were analyzed using the χ^2^ test or Fisher's exact test. Changes in the prevalence of categorical data between 2007 and 2017 were analyzed using logistic regression models. Continuous variables were expressed as the means and 95% CIs, and linear regression models were used to evaluate changes. To further examine the robustness of the results, we employed three models that adjusted for stepwise increases in risk factors for all participants. A two‐sided *p* value <.05 was considered statistically significant. All statistical analyses were performed using SUDAAN, version 10.0 (Research Triangle Institute).

## 3 RESULTS

### 3.1 Characteristics of the study participants

Table 1 presents the characteristics of the participants in each survey. Significant differences were observed in the mean age at survey, sex, income per year, education level, and family history of diabetes in participants between 2007 and 2017. Compared with participants from 2007, participants in 2017 were younger (44.8 years vs 43.8 years, *p* = .02) and there was a higher proportion of men (49.4% vs 50.2%, *p* = .0001). Higher income levels, education levels, and an increased likelihood of a family history of diabetes were observed in 2017 (*p* < .05 for all).

**TABLE 1:** Samples characteristics (weighted) by survey wave.

Characteristics	2007	2017	*p* for difference
No. of participants	46 239	73 340	
Mean age at survey (95% CI; years)	44.8 (44.5–45.1)	43.8 (42.9–44.6)	02
Sex			
Men	49.4 (49.3–49.6)	50.2 (49.9–50.6)	.0001
Women	50.6 (50.4–50.7)	49.8 (49.4–50.1)	
Residence			
Urban	45.8 (18.7–75.8)	52.0 (31.6–71.7)	.75
Rural	54.2 (24.3–81.4)	48.0 (28.3–68.4)	
Ethnicity			
Han	86.4 (69.2–94.7)	95.5 (93.6–96.8)	.16
Non‐Han	13.6 (5.3–30.8)	4.5 (3.2–6.5)	
Income per year			
≤30 000 yuan	82.8 (77.7–87.0)	44.7 (38.1–51.6)	<.0001
<30 000 yuan	17.2 (13.0–22.3)	55.3 (48.5–61.9)	
Education			
Less than college	79.6 (72.5–85.3)	66.2 (59.2–72.7)	.01
College and above	20.4 (14.7–27.6)	33.8 (27.3–40.9)	
Current cigarette smoker	26.1 (23.5–28.8)	26.7 (25.5–27.9)	.68
Family history of diabetes	11.4 (8.7–15.0)	16.4 (13.9–19.2)	.03

*Note*: Values are percentages (95% CI) unless stated otherwise.

Abbreviation: CI, confidence interval.

### 3.2 Changes in the prevalence of diabetes

Table 2 presents changes in the weighted prevalence of diagnosed diabetes, undiagnosed diabetes, and total diabetes in mainland China. Compared with those in 2007, the prevalence of diagnosed diabetes (3.8% vs 6.3%, *p* = .0001) and total diabetes among the overall population (9.7% vs 11.7%, *p* = .005) was significantly higher in 2017. In addition, the estimated prevalence of undiagnosed diabetes was 5.9% (95% CI, 5.0%–6.8%) in 2007 and 5.4% (95% CI, 4.9%–5.9%) in 2017 (*p* = .7) (Table 2).

**TABLE 2:** Changes in prevalence of diagnosed diabetes, undiagnosed diabetes, and total diabetes between 2007 and 2017 in adults in China.

	Diagnosed diabetes				Undiagnosed diabetes				Total diabetes			
	2007	2017	Odds ratio (95% CI) for change	*p* value	2007	2017	Odds ratio (95% CI) for change	*p* value	2007	2017	Odds ratio (95% CI) for change	*p* value
Overall	3.8 (2.8–5.1)	6.3 (5.8–6.8)	1.67 (1.31–2.13)	.0001	5.9 (5.0–6.8)	5.4 (4.9–5.9)	0.96 (0.80–1.16)	.7	9.7 (8.1–11.5)	11.7 (11.0–12.4)	1.27 (1.08–1.50)	.005
Sex												
Men	4.1 (2.8–5.9)	6.7 (6.0–7.4)	1.58 (1.18–2.12)	.003	6.5 (5.5–7.7)	6.0 (5.5–6.6)	0.95 (0.77–1.17)	.64	10.6 (8.6–13.1)	12.7 (11.9–13.5)	1.22 (1.00–1.48)	.04
Women	3.5 (2.7–4.5)	5.9 (5.4–6.4)	1.77 (1.39–2.26)	<.0001	5.2 (4.5–6.1)	4.8 (4.3–5.3)	0.97 (0.81–1.18)	.79	8.7 (7.4–10.3)	10.7 (10.0–11.4)	1.33 (1.14–1.56)	.0007
Urbanization												
Urban	5.0 (4.2–5.9)	6.8 (6.3–7.5)	1.56 (1.31–1.86)	<.0001	6.3 (5.4–7.4)	5.0 (4.4–5.7)	0.85 (0.67–1.09)	.19	11.2 (10.5–12.0)	11.8 (11.0–12.7)	1.18 (1.04–1.34)	.009
Rural	2.8 (1.7–4.8)	5.7 (4.8–6.7)	1.82 (1.11–2.99)	.02	5.5 (4.0–7.4)	5.8 (4.9–6.9)	1.06 (0.74–1.54)	.74	8.3 (6.0–11.4)	11.5 (10.1–13.1)	1.37 (0.99–1.89)	.06
Age group (years)												
20–29	0.3 (0.1–0.6)	0.8 (0.5–1.4)	3.73 (1.31–10.58)	.01	1.6 (1.1–2.4)	0.8 (0.6–1.1)	0.58 (0.28–1.18)	.13	1.9 (1.4–2.6)	1.6 (1.1–2.4)	1.07 (0.52–2.22)	.85
30–39	1.2 (1.0–1.5)	2.6 (2.1–3.2)	1.99 (1.54–2.56)	<.0001	2.9 (2.4–3.5)	2.8 (2.4–3.2)	1.09 (0.78–1.54)	.6	4.1 (3.5–4.8)	5.4 (4.6–6.3)	1.40 (1.11–1.76)	.006
40–49	3.1 (2.3–4.3)	4.8 (4.1–5.6)	1.65 (1.23–2.20)	.001	6.1 (5.3–7.0)	5.8 (5.1–6.5)	0.97 (0.78–1.21)	.79	9.2 (7.9–10.7)	10.5 (9.6–11.5)	1.21 (1.01–1.45)	.04
50–59	5.6 (4.1–7.6)	10.6 (9.6–11.6)	1.81 (1.44–2.27)	<.0001	8.7 (7.4–10.1)	8.4 (7.6–9.2)	0.97 (0.81–1.16)	.74	14.3 (11.8–17.2)	18.9 (17.8–20.1)	1.34 (1.14–1.59)	.0009
60–69	8.8 (6.4–12.0)	14.9 (12.8–17.3)	1.67 (1.26–2.21)	.0006	10.4 (8.7–12.3)	10.6 (9.4–12.0)	1.05 (0.80–1.38)	.72	19.2 (15.9–23.0)	25.5 (23.3–27.9)	1.38 (1.12–1.70)	.003
≥70	10.6 (6.5–17.0)	16.5 (13.8–19.5)	1.63 (0.94–2.81)	.08	11.3 (8.1–15.6)	12.3 (11.2–13.5)	1.05 (0.74–1.47)	.8	21.9 (15.6–29.8)	28.9 (25.7–32.0)	1.39 (0.96–2.01)	.08

*Note*: Values are weighted percentages (95% confidence intervals) unless stated otherwise. Logistic models were adjusted for age, sex, urbanization, ethnicity, income level, education level, family history of diabetes, and smoking status from 2007 to 2017.

Abbreviation: CI, confidence interval.

### 3.3 Changes in the prevalence of prediabetes

Table 3 presents the changes in the weighted prevalence of IFG, IGT, and prediabetes in mainland China. Compared with those in 2007, the prevalence of IFG (2.7% vs 2.6%, *p* = .68), IGT (12.7% vs 12.5%, *p* = .95), and prediabetes (15.4% vs 15.1%, *p* = .79) remained unchanged among the overall population in 2017.

**TABLE 3:** Changes in prevalence of IFG, IGT, and prediabetes between 2007 and 2017 in adults in China.

	Impaired fasting glucose				Impaired glucose tolerance				Prediabetes			
	2007	2017	Odds ratio (95% CI) for change	*p* value	2007	2017	Odds ratio (95% CI) for change	*p* value	2007	2017	Odds ratio (95% CI) for change	*p* value
Overall	2.7 (1.8–3.9)	2.6 (2.1–3.1)	1.10 (0.69–1.76)	.68	12.7 (11.3–14.3)	12.5 (11.6–13.6)	1.01 (0.84–1.21)	.95	15.4 (13.5–17.4)	15.1 (14.2–16.1)	1.03 (0.85–1.24)	.79
Sex												
Men	3.1 (2.1–4.7)	3.2 (2.7–3.9)	1.11 (0.69–1.80)	.66	12.9 (11.7–14.2)	12.2 (11.2–13.2)	0.95 (0.80–1.14)	.61	16.0 (14.1–18.1)	15.4 (14.4–16.4)	0.99 (0.80–1.22)	.91
Women	2.2 (1.5–3.3)	1.9 (1.6–2.4)	1.09 (0.67–1.78)	.73	12.6 (10.8–14.7)	12.9 (11.8–14.2)	1.06 (0.85–1.32)	.62	14.8 (12.6–17.3)	14.9 (13.8–16.0)	1.07 (0.86–1.32)	.56
Urbanization												
Urban	2.2 (1.5–3.1)	2.1 (1.7–2.8)	1.17 (0.74–1.86)	.49	12.5 (10.7–14.5)	12.7 (11.2–14.5)	1.06 (0.82–1.36)	.67	14.7 (12.4–17.2)	14.9 (13.5–16.4)	1.08 (0.85–1.38)	.53
Rural	3.1 (1.8–5.3)	3.1 (2.2–4.2)	1.04 (0.53–2.06)	.91	12.9 (9.9–16.7)	12.3 (10.8–14.1)	0.95 (0.66–1.37)	.77	16.0 (12.5–20.2)	15.4 (13.4–17.6)	0.97 (0.67–1.39)	.85
Age group (years)												
20–29	1.8 (1.2–2.8)	0.9 (0.7–1.3)	0.67 (0.37–1.20)	.17	4.8 (3.9–5.9)	4.6 (3.6–5.8)	1.10 (0.77–1.56)	.6	6.6 (5.3–8.3)	5.5 (4.5–6.8)	0.99 (0.70–1.39)	.93
30–39	2.2 (1.4–3.5)	1.8 (1.6–2.1)	0.89 (0.51–1.56)	.68	8.4 (7.0–10.0)	8.9 (7.6–10.3)	1.10 (0.86–1.41)	.46	10.6 (8.5–13.2)	10.7 (9.5–12.1)	1.06 (0.80–1.40)	.7
40–49	2.8 (2.1–3.7)	3.0 (2.4–3.7)	1.17 (0.78–1.74)	.44	13.9 (11.4–16.7)	13.7 (12.3–15.3)	1.01 (0.78–1.30)	.95	16.6 (13.9–19.9)	16.7 (15.3–18.2)	1.04 (0.81–1.33)	.75
50–59	3.2 (2.1–4.9)	4.1 (3.4–4.9)	1.37 (0.87–2.15)	.17	16.3 (14.4–18.3)	16.8 (15.5–18.3)	1.05 (0.85–1.29)	.64	19.5 (17.6–21.6)	20.9 (19.5–22.4)	1.11 (0.93–1.34)	.24
60–69	2.9 (1.7–4.8)	4.1 (3.0–5.4)	1.56 (0.89–2.72)	.12	20.2 (18.2–22.3)	20.7 (18.9–22.5)	1.02 (0.86–1.23)	.78	23.1 (21.4–24.8)	24.7 (23.4–26.1)	1.11 (0.96–1.28)	.15
≥70	3.6 (1.6–8.1)	3.3 (2.5–4.4)	1.09 (0.44–2.71)	.85	22.6 (19.1–26.6)	22.8 (21.2–24.5)	0.96 (0.75–1.24)	.76	26.2 (22.6–30.3)	26.1 (24.5–27.9)	0.98 (0.75–1.27)	.86

*Note*: Values are weighted percentages (95% confidence intervals) unless stated otherwise. Logistic models were adjusted for age, sex, urbanization, ethnicity, income level, education level, family history of diabetes, and smoking status from 2007 to 2017.

Abbreviations: CI, confidence interval; IFG, impaired fasting glucose; IGT, impaired glucose tolerance.

### 3.4 Changes in the prevalence of awareness, treatment, and control of diabetes

Table 4 shows the changes in the weighted prevalence of awareness, treatment, and control of diabetes stratified by sex, age group, and location between the two surveys, with adjustment for age, sex, urbanization, ethnicity, income level, education level, smoking status, and family history of diabetes. Significant increases were consistently found across all sexes, age groups (except for age ≥ 70), and urbanization statuses for awareness of diabetes (39.4% vs 53.6%, *p* = .0004, in the overall population); however, no significant increase in the prevalence of treatment (34.1% vs 32.5%, *p* = .44) or control (31.1% vs 32.8%, *p* = .73) of diabetes in the general population were observed.

**TABLE 4:** Changes in prevalence of awareness, treatment, and control of diabetes between 2007 and 2017 in adults in China.

	Awareness				Treatment				Control			
	2007	2017	Odds ratio (95% CI) for change	*p* value	2007	2017	Odds ratio (95% CI) for change	*p* value	2007	2017	Odds ratio (95% CI) for change	*p* value
Overall	39.4 (33.4–45.7)	53.6 (50.3–56.9)	1.69 (1.28–2.22)	.0004	34.1 (29.7–38.9)	32.5 (29.9–35.2)	0.92 (0.74–1.15)	.44	31.1 (25.7–37.2)	32.8 (29.7–36.1)	1.06 (0.74–1.53)	.73
Sex												
Men	38.6 (31.3–46.5)	52.5 (48.4–56.6)	1.63 (1.17–2.28)	.005	32.1 (26.3–38.7)	32.7 (29.6–35.9)	1.00 (0.77–1.30)	.99	29.8 (22.3–38.7)	30.7 (27.2–34.4)	0.96 (0.58–1.59)	.88
Women	40.3 (34.9–45.9)	55.0 (52.0–57.9)	1.74 (1.33–2.29)	.0001	36.5 (32.2–41.0)	32.2 (29.2–35.3)	0.85 (0.64–1.12)	.24	32.5 (27.8–37.5)	35.4 (31.2–39.8)	1.18 (0.81–1.73)	.37
Urbanization												
Urban	44.1 (36.8–51.5)	57.7 (54.0–61.2)	1.69 (1.21–2.36)	.003	37.8 (31.1–45.0)	35.0 (31.4–38.8)	0.91 (0.64–1.29)	.59	29.7 (22.9–37.6)	35.3 (30.3–40.8)	1.37 (0.89–2.08)	.15
Rural	34.0 (23.6–46.3)	49.2 (43.8–54.6)	1.71 (1.00–2.94)	.05	29.9 (22.1–39.1)	29.6 (24.8–35.0)	0.94 (0.58–1.51)	.78	33.1 (22.8–45.4)	29.4 (27.2–31.8)	0.82 (0.45–1.49)	.5
Age group (years)												
20–29	15.1 (6.8–30.2)	52.1 (43.4–60.7)	6.76 (2.64–17.35)	.0002	11.6 (3.9–29.8)	17.2 (10.5–26.8)	0.95 (0.27–3.29)	.93	30.0 (5.8–74.8)	66.3 (33.1–88.6)	13.35 (0.54–327.53)	.11
30–39	29.6 (24.3–35.4)	48.1 (43.4–52.8)	1.87 (1.22–2.85)	.005	24.6 (19.4–30.8)	17.8 (14.0–22.4)	0.67 (0.43–1.04)	.07	37.0 (26.4–49.1)	37.9 (30.3–46.1)	0.73 (0.22–2.40)	.59
40–49	33.9 (27.2–41.3)	45.5 (39.8–51.2)	1.73 (1.23–2.44)	.002	27.2 (22.8–32.0)	25.7 (22.4–29.4)	0.96 (0.74–1.25)	.75	31.2 (24.0–39.4)	27.4 (22.4–32.9)	0.65 (0.37–1.13)	.13
50–59	39.5 (33.6–45.7)	55.9 (52.0–59.6)	1.80 (1.40–2.31)	<.0001	35.5 (30.3–41.0)	36.6 (33.8–39.5)	1.01 (0.84–1.22)	.87	26.2 (19.6–34.2)	30.9 (27.1–35.0)	1.29 (0.82–2.04)	.27
60–69	45.8 (37.8–54.0)	58.4 (53.2–63.5)	1.54 (1.05–2.27)	.03	40.6 (34.1–47.4)	38.7 (34.6–43.1)	0.86 (0.63–1.17)	.32	32.5 (26.3–39.5)	30.6 (26.3–35.3)	1.04 (0.69–1.56)	.86
≥70	48.5 (36.3–61.0)	57.2 (52.7–61.7)	1.40 (0.77–2.53)	.26	42.7 (32.6–53.4)	37.1 (31.8–42.7)	0.72 (0.44–1.19)	.19	33.8 (19.8–51.3)	38.4 (32.5–44.6)	1.22 (0.55–2.72)	.62

*Note*: Values are weighted percentages (95% confidence intervals) unless stated otherwise. Logistic models were adjusted for age, sex, urbanization, ethnicity, income level, education level, family history of diabetes, and smoking status from 2007 to 2017.

Abbreviation: CI, confidence interval.

### 3.5 Changes in risk factor control rates

**FIGURE 1:**
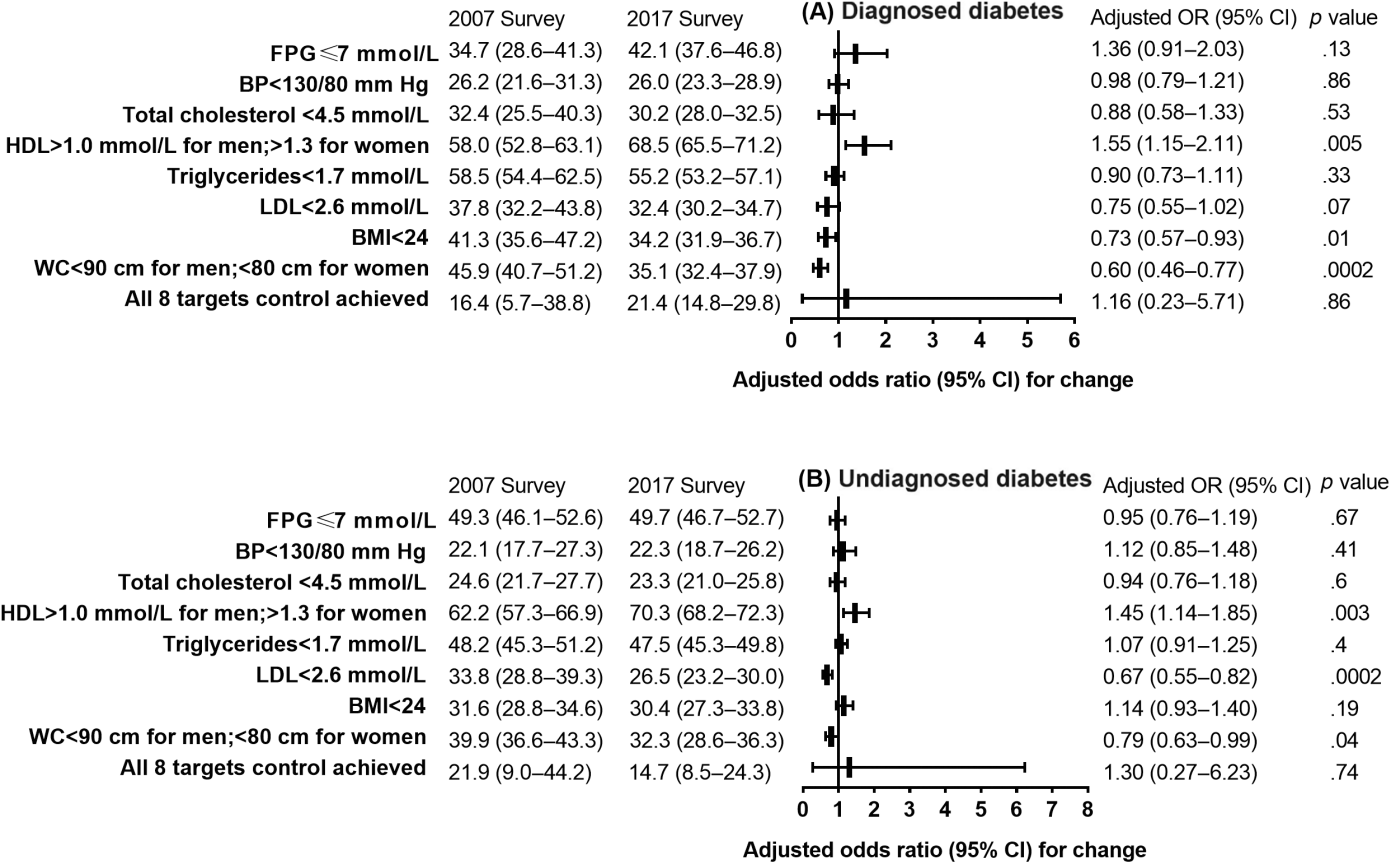
Changes in the proportion of risk factor control goals achieved among Chinese adults with (A) diagnosed diabetes and (B) undiagnosed diabetes. Diagnosed diabetes was defined as having self‐report of diabetes diagnosis by a physician or other health professional. Undiagnosed diabetes was defined as a fasting plasma glucose ≥7.0 mmol/L or 2 h plasma glucose after an oral glucose tolerance test ≥11.1 mmol/L among participants without self‐reported diabetes. Logistic models were adjusted for age, sex, urbanization, ethnicity, income level, education level, family history of diabetes, and smoking status from 2007 to 2017. BMI, body mass index; BP, blood pressure; CI, confidence interval; FPG, fasting plasma glucose; HDL‐C, high‐density lipoprotein; LDL‐C, low‐density lipoprotein; OR, odds ratio; WC, waist circumference.

Figure 1 illustrates the changes in the proportion of Chinese adults with diagnosed diabetes and undiagnosed diabetes who achieved risk factor control goals between 2007 and 2017. The estimated proportion of adults with diagnosed diabetes who achieved the HDL‐C control target significantly increased from 58.0% to 68.5% (*p* = .005) in this time frame, but the achievement of BMI and WC control targets decreased from 41.3% to 34.2% (*p* = .005) and from 45.9% to 35.1% (*p* = .0002), respectively. Among adults with diagnosed diabetes, the estimated proportion of achieved blood pressure, total cholesterol, triglycerides, and LDL‐C control targets did not change significantly overall (*p* > .05 for all). For adults with undiagnosed diabetes, the estimated proportion of achieved HDL‐C control targets significantly increased from 62.2% to 70.3% over 10 years (*p* = .003), but the achievement of LDL‐C and WC control targets decreased from 33.8% to 26.5% (*p* = .0002) and from 39.9% to 32.3% (*p* = .04), respectively.

### 3.6 Changes in cardiometabolic risk factors

Supplementary Table S2 shows the changes in the mean BMI, WC, systolic blood pressure (SBP), diastolic blood pressure, total cholesterol, HDL‐C, LDL‐C, triglycerides, and LAP index score among the overall population and the population with diagnosed diabetes, population with undiagnosed diabetes, and total diabetic population between the two surveys. The mean WC, SBP, HDL‐C, LDL‐C, and LAP index score significantly increased among all subgroups in 2017.

### 3.7 Sensitivity analysis

Sensitivity analyses for changes in the prevalence of various conditions are provided in the Supplementary Information. Overall, the prevalence of diabetes and prediabetes as well as the awareness, treatment, and control of diabetes remained stable according to three logistic regression models with progressive increases in the number of adjusted risk factors in the overall population (Supplementary Tables S3–S5).

## 4 DISCUSSION

In the current national representative study, we found that after adjusting for confounding demographic factors the weighted prevalence of diagnosed diabetes and total diabetes increased among adults in mainland China between 2007 and 2017. In addition, awareness of diabetes increased during this time span. We also found that the weighted prevalence of prediabetes as well as the rates of treatment and control of diabetes remained unchanged from 2007 to 2017. The estimated proportion of adults with diagnosed diabetes who achieved all eight risk factor control goals did not improve in 2017.

This study extends prior findings by providing estimates over a longer interval that were adjusted by confounding demographic factors and evaluating changes in known diabetes‐related risk factors as well as providing previously unreported changes in the control of risk factors for diabetes in China, based on nationally representative surveys with consistent study designs. Similar to a previous study, this work also documented an increased prevalence of diabetes among Chinese adults, which may be associated with many individual and contextual factors.^4^ First, the survival time of individuals with diabetes is prolonged due to the development of advanced treatments and antidiabetic drugs.^19^ Second, the prevalence of hypertension and obesity, especially abdominal obesity, increased significantly during this period; these are important risk factors for progression to diabetes.^13^ Third, an increased incidence of diabetes among young individuals may have contributed to an increased prevalence of diabetes in adults over the next 10 years.^5^ In addition, an increased prevalence of diagnosed diabetes among individuals aged 20–39 may have been associated with increased awareness of diabetes and active or physical screening. Higher consumption of processed and packaged foods and beverages among young adults may have contributed to the increases in prevalence.^20^

Notably, this study found that awareness of diabetes has improved over the past decade. In contrast, a previous study reported that awareness of diabetes remained unchanged from that in 2013.^4^ This difference may be explained by differences in the duration of the observation period. Improvements in awareness are likely attributed to the concerted effort of the Chinese Diabetes Society's Diabetes Prevention and Management Program (Bluelight Action), which was implemented in 2010.^21^ In addition, more widespread screening for chronic noncommunicable diseases and conventional physical examinations is reflected in our findings of an increased prevalence of diagnosed diabetes.

The stagnation in the rates of treatment and control is a crucial problem that cannot be ignored. Given the increased prevalence of diabetes, lack of change in the prevalence of prediabetes and the large population in China, diabetes‐related mortality and the economic burden will continue to increase, especially with an increasingly aging population.^5^ Improvements in the control of risk factors such as blood pressure, blood glucose levels, and blood lipid levels among adults with diabetes are critical to forecast population‐level complications and guide prevention efforts. Although the proportion of Chinese adults with diagnosed diabetes who achieved HDL‐C control targets improved, the control of other risk factors did not change significantly; moreover, the proportion of adults with diagnosed diabetes who achieved BMI and WC control targets decreased. In addition, the proportion of individuals with undiagnosed diabetes who achieved LDL‐C control targets decreased, further suggesting that there is still a long way to go in achieving comprehensive management of diabetes.

From a public health perspective, this study once again confirms the increasing trend of diabetes prevalence in China. Comprehensive action is needed to address the health, economic and social dimensions of the increasing national burden of diabetes. Emphasizing a healthy lifestyle to reduce the prevalence of abdominal obesity and thereby reduce risk factors for diabetes is highly recommended. From a clinical standpoint, achieving adequate control of risk factors for cardiovascular disease in diabetic patients remains challenging in China despite compelling evidence that adequate control of risk factors can delay or prevent cardiovascular disease in patients with diabetes.^22^ Inadequate control of diabetes may likely contribute to increases in diabetes‐related mortality. Therefore, policymakers should invest in clinical research related to diabetes control and treatment and implement established, cost‐effective interventions that target patients with diabetes. Furthermore, weight management remains an important aspect of diabetes control.

The two included surveys have potential limitations, some of which have been mentioned in previous studies.^11^, ^12^ First, they did not assess dietary intake, alcohol consumption, or physical activity. Therefore, we were not able to determine the associations between these factors and changes in the prevalence of diabetes. Second, although the survey staff were highly trained, their efficacy or skill level may have resulted in some misclassification errors. In addition, the limitation of the current analysis also warrants discussion. The first study did not measure glycated hemoglobin in participants, which limited our ability to compare the prevalence of diabetes by using the diagnostic criteria of the American Diabetes Association.

The prevalence of total diabetes significantly increased among adults in mainland China after adjustment for confounding demographic factors between 2007 and 2017. The awareness of diabetes improved over this period. However, more established and effective interventions and clinical strategies are urgently required to offset the potential burden due to lack of improvement in the rates of treatment and control of diabetes.

## AUTHOR CONTRIBUTION

Chenye Jin, Yaxin Lai, and Yongze Li contributed equally to this paper. Zhongyan Shan and Weiping Teng are joint corresponding authors. Chenye Jin, Yaxin Lai, Yongze Li, Zhongyan Shan, and Weiping Teng conceived and designed the study. Di Teng, Wenying Yang, Zhongyan Shan, and Weiping Teng supervised the study. Yongze Li performed the statistical analysis. The Thyroid Disorders, Iodine Status and Diabetes Epidemiological Survey Group conducted the epidemiological survey. All authors contributed to acquisition, analysis, or interpretation of data. Chenye Jin and Yongze Li drafted the manuscript. All authors revised the report and approved the final version before submission. Zhongyan Shan and Weiping Teng are the guarantors and attest that all listed authors meet authorship criteria and that no others meeting the criteria have been omitted.

## FUNDING INFORMATION

This work is supported by the Clinical Research Fund of the Chinese Medical Association (Grant No. 15010010589), the National Natural Science Foundation of China (Grant No. 82000753), and the China Postdoctoral Science Foundation (Grant No. 2021MD703910 and 2023T160724). The funder of the study had no role in the study design, data collection, data analysis, data interpretation, or the writing of the report. The corresponding author had full access to all the data in the study and had final responsibility for the decision to submit for publication.

## CONFLICT OF INTEREST STATEMENT

All authors declare no competing interests.

## Supporting information


**Figure S1.** Flow chart depicting survey design.
**Table S1.** Diagnostic criteria for diabetes related disorders.
**Table S2.** Adjusted changes in mean body mass index (BMI), waist circumference (WC), blood pressure, total, low density lipoprotein (LDL‐C), high density lipoprotein cholesterol (HDL‐C), triglycerides, and lipid accumulation product (LAP) index over the course of 10 years in adults in mainland China.
**Table S3.** Odds ratio (95% CI) for changes in weighted prevalence of diagnosed diabetes, undiagnosed diabetes, and total diabetes between 2007 and 2017 in adults in China by subgroups.
**Table S4.** Odds ratio (95% CI) for changes in weighted prevalence of impaired fasting glucose, impaired glucose tolerance, and prediabetes between 2007 and 2017 in adults in China by subgroups.
**Table S5.** Odds ratio (95% CI) for changes in weighted prevalence of awareness, treatment, and control of diabetes between 2007 and 2017 in adults in China by subgroups.

## Data Availability

All data will be made available on request to the corresponding author.
