# Supplementary material for: Changes in the prevalence of diabetes and control of risk factors for diabetes among Chinese adults from 2007 to 2017: An analysis of repeated national cross‐sectional surveys
Source: J Diabetes. 2023 Nov 5;16(2):e13492. doi: 10.1111/1753-0407.13492 (PMC10859318; doi:10.1111/1753-0407.13492)
Supplement: Supplementary file 1 — Figure S1. Flow chart depicting survey design. Table S1. Diagnostic criteria for diabetes related disorders. Table S2. Adjusted changes in mean body mass index (BMI), waist circumference (WC), blood pressure, total, low density lipoprotein (LDL‐C), high density lipoprotein cholesterol (HDL‐C), triglycerides, and lipid accumulation product (LAP) index over the course of 10 years in adults in mainland China. Table S3. Odds ratio (95% CI) for changes in weighted prevalence of diagnosed diabetes, undiagnosed diabetes, and total diabetes between 2007 and 2017 in adults in China by subgroups. Table S4. Odds ratio (95% CI) for changes in weighted prevalence of impaired fasting glucose, impaired glucose tolerance, and prediabetes between 2007 and 2017 in adults in China by subgroups. Table S5. Odds ratio (95% CI) for changes in weighted prevalence of awareness, treatment, and control of diabetes between 2007 and 2017 in adults in China by subgroups. [file JDB-16-e13492-s001.docx]

**SUPPLEMENTAL MATERIAL**

**Supplementary Figure 1. Flowchart depicting survey design**

Supplementary Figure Legends: For sampling process of the China National Diabetes and Metabolic Disorder Study, in the first-stage, 12 provinces and autonomous regions were selected from all 6 geographic regions in mainland China, in addition to the municipalities of Beijing and Shanghai. One midsize city (population 200,000-1,000,000), one developed and one underdeveloped county, which were at approximately the 67th and 33rd percentiles of gross domestic product (GDP) per capita among all counties within each province, respectively, were selected, plus the provincial capitals. In the second-stage, one to four urban districts from Beijing, Shanghai, and each provincial capital and a midsize city were randomly selected (a total of 76 urban districts). Two rural districts were randomly selected from each village and 4 rural districts were randomly selected from the Beijing and Shanghai countryside (a total of 56 rural districts). In the third-stage, two urban residential communities or rural residential communities were randomly selected from each urban city district and rural township district, respectively. At the final stage, eligible individuals from the local resident registration list who met the inclusion criteria were randomly selected according to age-sex-location composition among populations from China’s 2006 national census data. For sampling process of the Thyroid Disorders, Iodine Status and Diabetes Epidemiological Survey, at the first stage, one city was selected from each province in all 31 provinces of mainland China. Finally, 31 cities were selected and divided into developed, developing and underdeveloped cities, based on gross domestic product per capita, concentration of commercial resources, the extent to which a city serves as a commercial hub, vitality of residents, diversity of lifestyle and future dynamism. One county was randomly selected from each city. At the second stage, one urban district was randomly selected from each city (a total of 31 urban districts) and one rural district was randomly selected from each village (a total of 31 rural districts). At the third stage, two urban residential communities or rural residential communities were randomly selected from each urban city district and rural township district, respectively. At the final stage, eligible individuals from the local resident registration list who met the inclusion criteria were randomly selected according to age-sex-location composition among the population from China’s 2010 national census data.


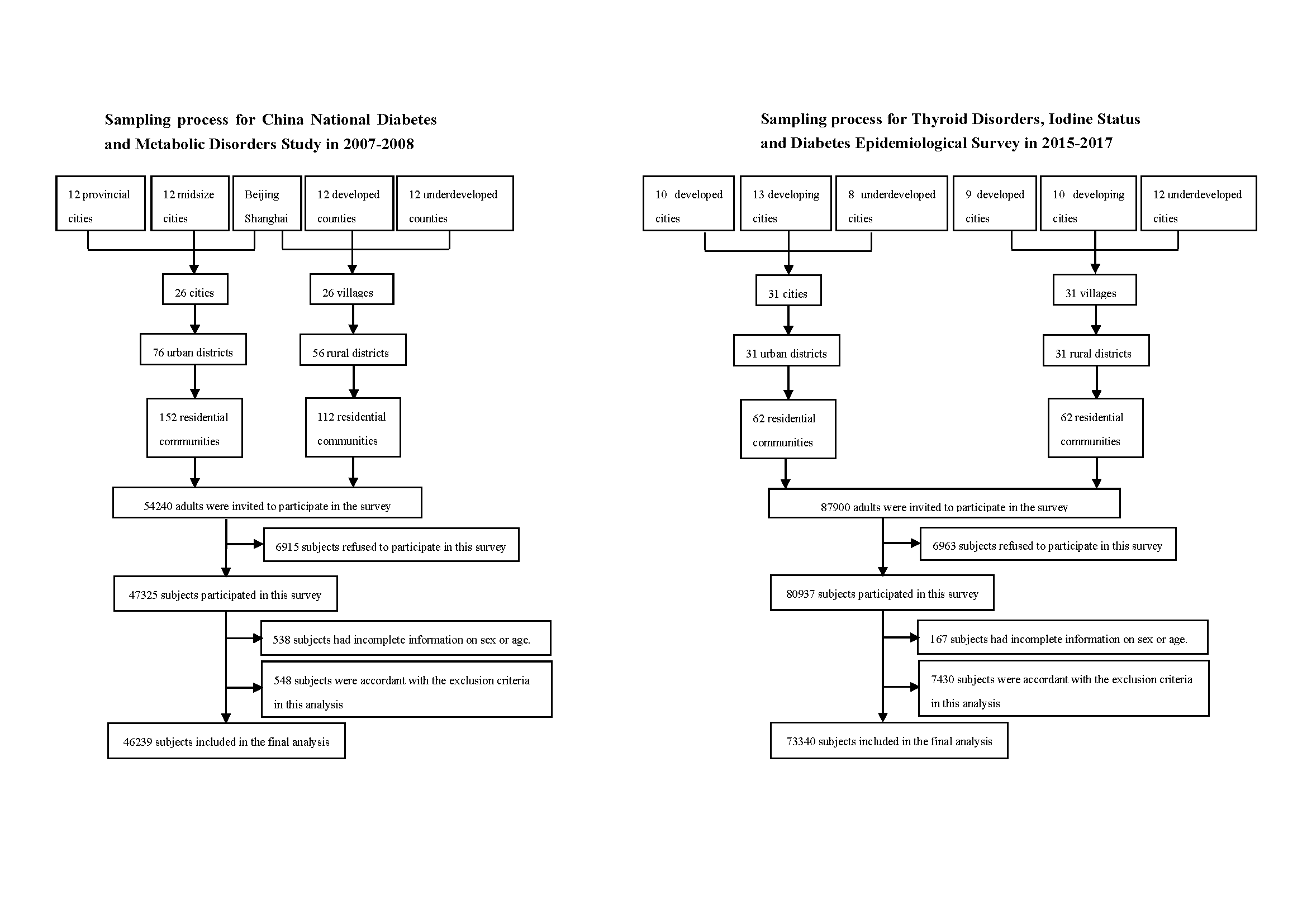


**Supplementary Table 1. Diagnostic criteria for diabetes related disorders**

| No. | Disorders | Diagnostic criteria |
| --- | --- | --- |
| 1 | Diagnosed diabetes | A self-reported diagnosis that was determined previously by a healthcare professional |
| 2 | Undiagnosed diabetes | A fasting plasma glucose ≥7.0 mmol/L or two hour plasma glucose after an oral glucose tolerance test ≥11.1 mmol/L among participants without self-reported diabetes |
| 3 | Total diabetes | Sum of number of patients with diagnosed diabetes and the number of patients with undiagnosed diabetes |
| 4 | Impaired fasting glucose | A fasting plasma glucose 6.1 mmol/L to 6.9 mmol/L, and two hour plasma glucose after an oral glucose tolerance test <7.8 mmol/L among participants without diabetes |
| 5 | Impaired glucose tolerance | A fasting plasma glucose <7.0 mmol/L, and two hour plasma glucose after an oral glucose tolerance test 7.8 mmol/L to 11.0 mmol/L among participants without diabetes |
| 6 | Prediabetes | A fasting plasma glucose 6.1 mmol/L to 6.9 mmol/L, and two hour plasma glucose after an oral glucose tolerance test <7.8 mmol/L, or fasting plasma glucose <7.0 mmol/L, and two hour plasma glucose after an oral glucose tolerance test 7.8 mmol/L to 11.0 mmol/L among participants without diabetes |
| 7 | Awareness of diabetes | The proportion of individuals with self-reported, physician-diagnosed diabetes among all patients with diabetes |
| 8 | Treatment of diabetes | The proportion of individuals taking drugs for diabetes among those diagnosed with the disease |
| 9 | Control of diabetes | The proportion of individuals with a fasting plasma glucose ≤7.0 mmol/L among patients with diabetes who were taking medication |

**Supplementary Table 2. Adjusted changes in mean body mass index (BMI), waist circumference (WC), blood pressure, total, low density lipoprotein (LDL-C), high density lipoprotein cholesterol (HDL-C), triglycerides, and lipid accumulation product (LAP) index over the course of 10 years in adults in mainland China.**

| Indicator | 2007 | 2017 | Adjusted changes | P value |
| --- | --- | --- | --- | --- |
| Overall population |  |  |  |  |
| BMI | 23.71 (23.38-24.05) | 24.09 (23.96-24.21) | 0.21 (-0.01 to 0.42) | 0.05 |
| WC | 80.71 (79.54-81.87) | 83.45 (82.70-84.20) | 2.47 (1.30 to 3.64) | 0.0001 |
| SBP | 121.70 (120.10-123.29) | 126.60 (125.25-127.95) | 5.33 (3.94 to 6.71) | <0.0001 |
| DBP | 77.55 (76.33-78.78) | 78.55 (77.41-79.69) | 0.80 (-0.76 to 2.37) | 0.31 |
| Total cholesterol | 4.71 (4.63-4.79) | 4.80 (4.75-4.85) | 0.08 (0.001 to 0.16) | 0.048 |
| Triglycerides | 1.56 (1.48-1.64) | 1.59 (1.54-1.64) | 0.001 (-0.09 to 0.10) | 0.93 |
| HDL-C | 1.30 (1.26-1.33) | 1.47 (1.45-1.48) | 0.19 (0.14 to 0.23) | <0.0001 |
| LDL-C | 2.66 (2.56-2.77) | 2.85 (2.79-2.91) | 0.18 (0.07 to 0.29) | 0.002 |
| LAP index | 33.67 (30.67-36.67) | 38.57 (36.79-40.36) | 4.15 (1.06 to 7.25) | 0.01 |
| Diagnosed diabetes |  | |  |  |
| BMI | 24.96 (24.55-25.36) | 25.50 (25.31-25.69) | 0.52 (0.08 to 0.96) | 0.02 |
| WC | 86.65 (85.49-87.81) | 88.83 (88.29-89.36) | 2.18 (0.94 to 3.43) | 0.0009 |
| SBP | 133.92 (131.58-136.26) | 136.77 (134.90-138.63) | 3.12 (1.22 to 5.01) | 0.002 |
| DBP | 80.93 (79.27-82.58) | 81.45 (80.56-82.33) | 0.05 (-1.53 to 1.63) | 0.95 |
| Total cholesterol | 4.95 (4.78-5.12) | 5.09 (5.04-5.14) | 0.15 (-0.05 to 0.34) | 0.15 |
| Triglycerides | 1.96 (1.86-2.05) | 2.10 (2.00-2.20) | 0.10 (-0.05 to 0.25) | 0.19 |
| HDL-C | 1.23 (1.18-1.29) | 1.36 (1.34-1.38) | 0.13 (0.07 to 0.20) | 0.0001 |
| LDL-C | 2.91 (2.80-3.02) | 3.04 (2.98-3.10) | 0.16 (0.03 to 0.28) | 0.02 |
| LAP index | 50.96 (48.22-53.71) | 60.02 (56.37-63.67) | 8.01 (2.95 to 13.06) | 0.003 |
| Undiagnosed diabetes | | | |  |
| BMI | 25.85 (25.51-26.19) | 25.95 (25.69-26.20) | -0.28 (-0.77 to 0.21) | 0.26 |
| WC | 87.48 (86.62-88.34) | 89.65 (88.68-90.61) | 1.56 (0.29 to 2.82) | 0.02 |
| SBP | 133.98 (132.18-135.77) | 138.53 (136.33-140.74) | 3.53 (1.04 to 6.02) | 0.007 |
| DBP | 83.11 (82.33-83.89) | 84.28 (82.44-86.13) | 0.78 (-1.35 to 2.91) | 0.46 |
| Total cholesterol | 5.15 (5.08-5.23) | 5.31 (5.24-5.37) | 0.13 (0.01 to 0.24) | 0.03 |
| Triglycerides | 2.11 (1.98-2.24) | 2.35 (2.25-2.44) | 0.19 (0.02 to 0.37) | 0.03 |
| HDL-C | 1.28 (1.24-1.33) | 1.38 (1.35-1.40) | 0.11 (0.06 to 0.16) | <0.0001 |
| LDL-C | 2.92 (2.82-3.02) | 3.16 (3.08-3.25) | 0.26 (0.14 to 0.38) | 0.0001 |
| LAP index | 56.98 (53.03-60.93) | 68.46 (65.04-71.87) | 9.72 (3.43 to 16.01) | 0.003 |
| Total diabetes |  | |  |  |
| BMI | 25.50 (25.29-25.70) | 25.71 (25.54-25.88) | 0.01 (-0.34 to 0.36) | 0.97 |
| WC | 87.16 (86.50-87.81) | 89.21 (88.53-89.89) | 1.73 (0.67 to 2.78) | 0.002 |
| SBP | 133.96 (132.71-135.20) | 137.58 (135.74-139.42) | 3.09 (1.24 to 4.94) | 0.002 |
| DBP | 82.25 (81.63-82.87) | 82.76 (81.43-84.09) | 0.12 (-1.35 to 1.60) | 0.87 |
| Total cholesterol | 5.08 (4.97-5.18) | 5.19 (5.15-5.24) | 0.11 (-0.02 to 0.25) | 0.11 |
| Triglycerides | 2.05 (1.96-2.15) | 2.22 (2.15-2.29) | 0.12 (-0.02 to 0.27) | 0.08 |
| HDL-C | 1.26 (1.21-1.31) | 1.37 (1.35-1.39) | 0.12 (0.06 to 0.17) | 0.0001 |
| LDL-C | 2.92 (2.83-3.01) | 3.10 (3.04-3.16) | 0.20 (0.09 to 0.31) | 0.0007 |
| LAP index | 54.62 (52.00-57.24) | 63.94 (61.22-66.66) | 7.96 (2.98 to 12.95) | 0.002 |

Note: Values are means (95% confidence intervals). Regression models were adjusted for age, sex, urbanization, ethnicity, income level, education level, family history of diabetes, and smoking status from 2007 to 2017.

**Supplementary Table 3. Odds ratio (95% CI) for changes in weighted prevalence of diagnosed diabetes, undiagnosed diabetes, and total diabetes between 2007 and 2017 in adults in China by subgroups.**

|  | Diagnosed diabetes | | | Undiagnosed diabetes | | | Total diabetes | | |
| --- | --- | --- | --- | --- | --- | --- | --- | --- | --- |
|  | Model 1^a^ | Model 2^b^ | Model 3^c^ | Model 1^a^ | Model 2^b^ | Model 3^c^ | Model 1^a^ | Model 2^b^ | Model 3^c^ |
| Overall | 1.69 (1.23-2.32)* | 1.75 (1.25-2.46)* | 1.67 (1.31-2.13)* | 0.92 (0.76-1.11) | 0.93 (0.78-1.13) | 0.96 (0.80-1.16) | 1.24 (1.01-1.52)* | 1.29 (1.03-1.61)* | 1.27 (1.08-1.50)* |
| Sex |  |  |  |  |  |  |  |  |  |
| Men | 1.67 (1.12-2.49)* | 1.75 (1.16-2.66)* | 1.58 (1.18-2.12)* | 0.92 (0.75-1.13) | 0.94 (0.77-1.16) | 0.95 (0.77-1.17) | 1.22 (0.96-1.56) | 1.28 (0.99-1.67) | 1.22 (1.00-1.48)* |
| Women | 1.71 (1.31-2.25)* | 1.75 (1.29-2.36)* | 1.77 (1.39-2.26)* | 0.92 (0.76-1.12) | 0.92 (0.75-1.13) | 0.97 (0.81-1.18) | 1.25 (1.03-1.52)* | 1.28 (1.03-1.60)* | 1.33 (1.14-1.56)* |
| Urbanization |  |  |  |  |  |  |  |  |  |
| Urban | 1.41 (1.15-1.73)* | 1.58 (1.31-1.92)* | 1.56 (1.31-1.86)* | 0.79 (0.64-0.97) | 0.84 (0.67-1.06) | 0.85 (0.67-1.09) | 1.06 (0.95-1.19) | 1.18 (1.05-1.34)* | 1.18 (1.04-1.34)* |
| Rural | 2.06 (1.15-3.68)* | 1.98 (1.11-3.55)* | 1.82 (1.11-2.99)* | 1.07 (0.74-1.54) | 1.03 (0.71-1.49) | 1.06 (0.74-1.54) | 1.43 (0.98-2.08) | 1.39 (0.95-2.04) | 1.37 (0.99-1.89) |
| Age group |  |  |  |  |  |  |  |  |  |
| 20-29 | 2.96 (1.23-7.09)* | 3.35 (1.37-8.22)* | 3.73 (1.31-10.58)* | 0.47 (0.28-0.80)* | 0.51 (0.30-0.87)* | 0.58 (0.28-1.18) | 0.85 (0.51-1.41) | 0.93 (0.55-1.59) | 1.07 (0.52-2.22) |
| 30-39 | 2.16 (1.61-2.91)* | 2.20 (1.63-2.98)* | 1.99 (1.54-2.56)* | 0.97 (0.75-1.25) | 0.97 (0.75-1.26) | 1.09 (0.78-1.54) | 1.33 (1.05-1.68)* | 1.35 (1.07-1.71)* | 1.40 (1.11-1.76)* |
| 40-49 | 1.56 (1.09-2.25)* | 1.57 (1.09-2.27)* | 1.65 (1.23-2.20)* | 0.94 (0.77-1.15) | 0.94 (0.77-1.15) | 0.97 (0.78-1.21) | 1.16 (0.96-1.41) | 1.17 (0.96-1.42) | 1.21 (1.01-1.45)* |
| 50-59 | 1.98 (1.40-2.79)* | 1.92 (1.37-2.71)* | 1.81 (1.44-2.27)* | 0.96 (0.79-1.17) | 0.94 (0.78-1.14) | 0.97 (0.81-1.16) | 1.40 (1.11-1.77)* | 1.37 (1.09-1.71)* | 1.34 (1.14-1.59)* |
| 60-69 | 1.82 (1.24-2.68)* | 1.83 (1.25-2.68)* | 1.67 (1.26-2.21)* | 1.02 (0.81-1.29) | 1.03 (0.81-1.30) | 1.05 (0.80-1.38) | 1.44 (1.11-1.87)* | 1.45 (1.12-1.88)* | 1.38 (1.12-1.70)* |
| ≥70 | 1.66 (0.93-2.95) | 1.63 (0.93-2.87) | 1.63 (0.94-2.81) | 1.10 (0.75-1.62) | 1.04 (0.70-1.56) | 1.05 (0.74-1.47) | 1.44 (0.92-2.24) | 1.38 (0.89-2.14) | 1.39 (0.96-2.01) |

^a^ Model 1: Unadjusted.

^b^ Model 2: Adjusted for age and sex.

^c^ Model 3: Adjusted for age, sex, location, ethnicity, income level, education level, family history of diabetes, and smoking status.

^*^ indicates a *P*-value less than 0.05.

**Supplementary Table 4. Odds ratio (95% CI) for changes in weighted prevalence of impaired fasting glucose, impaired glucose tolerance, and prediabetes between 2007 and 2017 in adults in China by subgroups.**

|  | Impaired fasting glucose | | | Impaired glucose tolerance | | | Prediabetes | | |
| --- | --- | --- | --- | --- | --- | --- | --- | --- | --- |
|  | Model 1^a^ | Model 2^b^ | Model 3^c^ | Model 1^a^ | Model 2^b^ | Model 3^c^ | Model 1^a^ | Model 2^b^ | Model 3^c^ |
| Overall | 0.97 (0.62-1.53) | 0.99 (0.63-1.55) | 1.10 (0.69-1.76) | 0.98 (0.84-1.15) | 1.00 (0.84-1.20) | 1.01 (0.84-1.21) | 0.98 (0.83-1.16) | 1.00 (0.84-1.19) | 1.03 (0.85-1.24) |
| Sex |  |  |  |  |  |  |  |  |  |
| Men | 1.04 (0.65-1.66) | 1.06 (0.67-1.69) | 1.11 (0.69-1.80) | 0.93 (0.81-1.08) | 0.96 (0.82-1.13) | 0.95 (0.80-1.14) | 0.95 (0.81-1.13) | 0.98 (0.82-1.17) | 0.99 (0.80-1.22) |
| Women | 0.88 (0.55-1.40) | 0.89 (0.56-1.41) | 1.09 (0.67-1.78) | 1.03 (0.84-1.26) | 1.05 (0.84-1.31) | 1.06 (0.85-1.32) | 1.00 (0.82-1.23) | 1.02 (0.82-1.27) | 1.07 (0.86-1.32) |
| Urbanization |  |  |  |  |  |  |  |  |  |
| Urban | 0.99 (0.63-1.57) | 1.03 (0.65-1.63) | 1.17 (0.74-1.86) | 1.02 (0.81-1.28) | 1.09 (0.85-1.40) | 1.06 (0.82-1.36) | 1.02 (0.81-1.27) | 1.09 (0.85-1.39) | 1.08 (0.85-1.38) |
| Rural | 1.00 (0.52-1.91) | 0.99 (0.51-1.90) | 1.04 (0.53-2.06) | 0.95 (0.67-1.33) | 0.92 (0.66-1.30) | 0.95 (0.66-1.37) | 0.95 (0.69-1.32) | 0.93 (0.67-1.31) | 0.97 (0.67-1.39) |
| Age group |  |  |  |  |  |  |  |  |  |
| 20-29 | 0.50 (0.29-0.86)* | 0.52 (0.30-0.90)* | 0.67 (0.37-1.20) | 0.95 (0.67-1.34) | 1.00 (0.71-1.40) | 1.10 (0.77-1.56) | 0.82 (0.59-1.14) | 0.86 (0.62-1.19) | 0.99 (0.70-1.39) |
| 30-39 | 0.81 (0.50-1.31) | 0.81 (0.50-1.32) | 0.89 (0.51-1.56) | 1.07 (0.82-1.38) | 1.07 (0.82-1.40) | 1.10 (0.86-1.41) | 1.01 (0.76-1.34) | 1.02 (0.76-1.35) | 1.06 (0.80-1.40) |
| 40-49 | 1.07 (0.75-1.54) | 1.07 (0.75-1.54) | 1.17 (0.78-1.74) | 0.99 (0.77-1.28) | 0.99 (0.77-1.28) | 1.01 (0.78-1.30) | 1.00 (0.79-1.28) | 1.01 (0.79-1.28) | 1.04 (0.81-1.33) |
| 50-59 | 1.26 (0.78-2.04) | 1.24 (0.76-2.02) | 1.37 (0.87-2.15) | 1.04 (0.88-1.24) | 1.03 (0.87-1.23) | 1.05 (0.85-1.29) | 1.09 (0.93-1.27) | 1.08 (0.92-1.26) | 1.11 (0.93-1.34) |
| 60-69 | 1.41 (0.76-2.59) | 1.41 (0.77-2.58) | 1.56 (0.89-2.72) | 1.03 (0.87-1.22) | 1.03 (0.87-1.22) | 1.02 (0.86-1.23) | 1.10 (0.97-1.23) | 1.10 (0.97-1.24) | 1.11 (0.96-1.28) |
| ≥70 | 0.92 (0.37-2.25) | 1.02 (0.45-2.32) | 1.09 (0.44-2.71) | 1.01 (0.80-1.28) | 1.00 (0.78-1.27) | 0.96 (0.75-1.24) | 0.99 (0.80-1.24) | 1.00 (0.79-1.26) | 0.98 (0.75-1.27) |

^a^ Model 1: Unadjusted.

^b^ Model 2: Adjusted for age and sex.

^c^ Model 3: Adjusted for age, sex, location, ethnicity, income level, education level, family history of diabetes, and smoking status.

^*^ indicates a *P*-value less than 0.05.

**Supplementary Table 5. Odds ratio (95% CI) for changes in weighted prevalence of awareness, treatment, and control of diabetes between 2007 and 2017 in adults in China by subgroups.**

|  | Awareness of diabetes | | | Treatment of diabetes | | | Control of diabetes | | |
| --- | --- | --- | --- | --- | --- | --- | --- | --- | --- |
|  | Model 1^a^ | Model 2^b^ | Model 3^c^ | Model 1^a^ | Model 2^b^ | Model 3^c^ | Model 1^a^ | Model 2^b^ | Model 3^c^ |
| Overall | 1.78 (1.33-2.39)* | 1.77 (1.32-2.38)* | 1.69 (1.28-2.22)* | 0.93 (0.73-1.18) | 0.91 (0.72-1.15) | 0.92 (0.74-1.15) | 1.08 (0.80-1.47) | 1.09 (0.80-1.48) | 1.06 (0.74-1.53) |
| Sex |  |  |  |  |  |  |  |  |  |
| Men | 1.76 (1.23-2.53)* | 1.75 (1.21-2.52)* | 1.63 (1.17-2.28)* | 1.03 (0.75-1.41) | 1.01 (0.73-1.40) | 1.00 (0.77-1.30) | 1.04 (0.68-1.60) | 1.03 (0.67-1.59) | 0.96 (0.58-1.59) |
| Women | 1.81 (1.40-2.35)* | 1.81 (1.40-2.33)* | 1.74 (1.33-2.29)* | 0.83 (0.65-1.05) | 0.80 (0.64-1.01) | 0.85 (0.64-1.12) | 1.14 (0.85-1.52) | 1.14 (0.85-1.52) | 1.18 (0.81-1.73) |
| Urbanization |  |  |  |  |  |  |  |  |  |
| Urban | 1.73 (1.24-2.42)* | 1.74 (1.25-2.42)* | 1.69 (1.21-2.36)* | 0.89 (0.63-1.25) | 0.87 (0.63-1.21) | 0.91 (0.64-1.29) | 1.29 (0.85-1.97) | 1.32 (0.86-2.01) | 1.37 (0.89-2.08) |
| Rural | 1.88 (1.08-3.28)* | 1.86 (1.08-3.20)* | 1.71 (1.00-2.94) | 0.99 (0.61-1.59) | 0.96 (0.61-1.53) | 0.94 (0.58-1.51) | 0.84 (0.50-1.43) | 0.84 (0.49-1.44) | 0.82 (0.45-1.49) |
| Age group |  |  |  |  |  |  |  |  |  |
| 20-29 | 6.14 (2.35-16.02)* | 6.04 (2.26-16.16)* | 6.76 (2.64-17.35)* | 1.57 (0.43-5.77) | 1.63 (0.49-5.46) | 0.95 (0.27-3.29) | 4.58 (0.43-49.37) | 4.21 (0.41-43.02) | 13.35 (0.54-327.53) |
| 30-39 | 2.21 (1.59-3.06)* | 2.26 (1.60-3.18)* | 1.87 (1.22-2.85)* | 0.66 (0.44-1.01) | 0.71 (0.46-1.10) | 0.67 (0.43-1.04) | 1.04 (0.57-1.89) | 1.02 (0.53-1.94) | 0.73 (0.22-2.40) |
| 40-49 | 1.63 (1.10-2.41)* | 1.64 (1.11-2.43)* | 1.73 (1.23-2.44)* | 0.93 (0.69-1.25) | 0.95 (0.71-1.28) | 0.96 (0.74-1.25) | 0.83 (0.53-1.30) | 0.80 (0.50-1.27) | 0.65 (0.37-1.13) |
| 50-59 | 1.94 (1.44-2.61)* | 1.94 (1.43-2.61)* | 1.80 (1.40-2.31)* | 1.05 (0.81-1.37) | 1.04 (0.80-1.36) | 1.01 (0.84-1.22) | 1.26 (0.83-1.91) | 1.26 (0.83-1.92) | 1.29 (0.82-2.04) |
| 60-69 | 1.66 (1.13-2.46)* | 1.66 (1.13-2.46)* | 1.54 (1.05-2.27)* | 0.93 (0.67-1.29) | 0.93 (0.67-1.29) | 0.86 (0.63-1.17) | 0.92 (0.63-1.33) | 0.94 (0.66-1.35) | 1.04 (0.69-1.56) |
| ≥70 | 1.42 (0.83-2.43) | 1.48 (0.87-2.52) | 1.40 (0.77-2.53) | 0.79 (0.49-1.29) | 0.83 (0.52-1.33) | 0.72 (0.44-1.19) | 1.22 (0.57-2.63) | 1.20 (0.58-2.50) | 1.22 (0.55-2.72) |

^a^ Model 1: Unadjusted.

^b^ Model 2: Adjusted for age and sex.

^c^ Model 3: Adjusted for age, sex, location, ethnicity, income level, education level, family history of diabetes, and smoking status.

^*^ indicates a *P*-value less than 0.05.
